# Characterization of genetically modified mice for phosphoglycerate mutase, a vitally-essential enzyme in glycolysis

**DOI:** 10.1371/journal.pone.0250856

**Published:** 2021-04-29

**Authors:** Takumi Mikawa, Eri Shibata, Midori Shimada, Ken Ito, Tomiko Ito, Hiroaki Kanda, Keiyo Takubo, Atsuyoshi Shimada, Matilde E. Lleonart, Nobuya Inagaki, Masayuki Yokode, Hiroshi Kondoh

**Affiliations:** 1 Geriatric Unit, Graduate School of Medicine, Kyoto University, Kyoto, Japan; 2 Department of Diabetes, Endocrinology and Nutrition, Graduate School of Medicine, Kyoto University, Kyoto, Japan; 3 Joint Faculty of Veterinary Science, Yamaguchi University, Yamaguchi, Japan; 4 Department of Pathology, Saitama Cancer Center, Saitama, Japan; 5 Department of Stem Cell Biology, Research Institute, National Center for Global Health and Medicine, Tokyo, Japan; 6 Faculty of Health Sciences, Kyorin University, Tokyo, Japan; 7 Department of Pathology, Hospital Vall de’Hebron, Barcelona, Spain; Kindai University: Kinki Daigaku, JAPAN

## Abstract

Glycolytic metabolism is closely involved in physiological homeostasis and pathophysiological states. Among glycolytic enzymes, phosphoglycerate mutase (PGAM) has been reported to exert certain physiological role *in vitro*, whereas its impact on glucose metabolism *in vivo* remains unclear. Here, we report the characterization of *Pgam1* knockout mice. We observed that homozygous knockout mice of *Pgam1* were embryonic lethal. Although we previously reported that both PGAM-1 and -2 affect global glycolytic profile of cancers *in vitro*, *in vivo* glucose parameters were less affected both in the heterozygous knockout of *Pgam1* and in *Pgam2* transgenic mice. Thus, the impact of PGAM on *in vivo* glucose metabolism is rather complex than expected before.

## Introduction

Glucose is utilized as energy source, from bacterias to higher eukaryotes. Glycolysis provides energy supply in the form of ATP through oxidation of carbon atoms in glucose. As it also supports the synthesis of macromolecules [1], glycolysis is evolutionally conserved as one of vitally essential metabolisms.

Dysregulation in glycolysis, either the pathological enhancement or impairment, is closely related to human disease states. For example, most of cancer cells display enhanced glycolytic feature, known as the Warburg effect. On the other hand, impaired glycolysis is much involved in dysfunction in various tissues and degenerative disorders [2,3]. In humans and other vertebrates, glucose is delivered to various tissues through circulating blood. Even during prolonged fasting (no calorie feeding), blood sugar levels are minimally maintained, which enables various tissues and cells, e.g. neurons in brain, to utilize glucose from circulating blood. The patients with impaired uptake of glucose into cells display higher glucose levels in blood, known as diabetes or impaired glucose metabolism. Indeed, diabetes are observed both in human glycolytic enzyme deficiencies [4,5] and in knockout (KO) mice for glycolytic enzymes [6–9].

Glycolytic enzyme phosphoglycerate mutase (PGAM) converts 3-phosphoglycerate into 2-phosphoglycerate as an isomerase [10]. Two isoforms of PGAM (PGAM1 and PGAM2) distribute to overlapping but distinct tissues *in vivo*. However, both isoforms display the significant similarity in their sequences (79% identity) and enzymatic activities *in vitro* [11–13].

Besides its role in glycolysis, PGAM would play a key role connecting glycolysis to physiological homeostasis. PGAM is post-translationally regulated by several modification, including the phosphorylation, acetylation and ubiquitination by environmental stimuli or under stress conditions [13–15]. Moreover, PGAM activity modulates mitochondrial function [16] and the pentose phosphate pathway [17], implicating its involvement in the defense against oxidative stress [12]. Noteworthy, both PGAM-1 and -2 support cancerous cell growth through its nonenzymatic function. PGAM interacts with Chk1 kinase under oncogenic Ras expression, followed by boosts of glycolytic profiles [18].

However, the global impacts of PGAM *in vivo* is still unclear. Here we characterized the profiles of glucose metabolisms in mice with ablation of *Pgam1* or global overexpression of *Pgam2 in vivo*. Homozygous knockout of *Pgam1* is embryonic lethal, while its global heterozygous knockout mice displayed comparable glucose parameters *in vivo*, compared to those in wild type. Moreover, global overexpression of *Pgam2* unlikely affects the *in vivo* profile for glucose metabolisms. Thus, the impact of PGAM on *in vivo* glucose metabolism is rather complex than expected before.

## Materials and methods

### Ethical statements

All procedures for animal experiments were approved and conducted in accordance with the principles and guidelines of the Animal Care and Use Committees of Kyoto University Graduate School of Medicine (Med Kyo 15556 and Med Kyo 15558).

### Generation of mouse models

*Pgam2*-Tg mice were generated previously [13]. *Pgam2-*Tg is a strain of transgenic C57BL/6 mice that overexpresses the *Pgam2* with a 3xFLAG tag under the cytomegalovirus immediate-to-early enhancer element and chicken *β-actin* promoter (*CAG*) [19].

Generation of the conditional knockout mouse of *Pgam1* is previously reported [18]. For the study of whole body *Pgam1 knockout* mice, *Pgam1*^*+/-*^ mice were generated by crossing between *Pgam1*^*flox/+*^ mice and *CAG*-*Cre* Tg mice, which ubiquitously express Cre recombinase under the *CAG* promoter. Subsequently, *Pgam1*^*+/-*^; CAG-Cre micewere crossed with wild-type mice and the genotypes of progenitor mice were analyzed by using PCR. Resulting *Pgam1*^*+/-*^ mice without CAG-Cre transgene were applied for further experiment.

### Mouse experiments

Intraperitoneal glucose tolerance test (IPGTT) was performed as previously described [20]. After 16 h of fasting, 1.5 g/kg body weight glucose was intraperitoneally injected into the mice. The blood glucose level of the mice was measured at 0, 15, 30, 60, and 120 min using a Glutest sensor (Sanwa Kagaku Kenkyusyo Co., Ltd. Aichi, Japan). For the high-fat diet study, 8-week-old male mice were fed diets with 60% of calories from fat (high-fat diet, Research Diets D12492) for 10 weeks. Body weight was measured every week. Finally, IPGTT was performed when the mice 18 weeks old. Blood tests for several biological markers were performed by Unitech Co. (Kashiwa, Japan). Muscle muss and body fat mass in mice were measured using magnetic resonance imaging (Latheta LCT-100, Hitachi).

### Measurement of glycolytic enzyme activity

PGAM enzymatic activity was measured as previously described [18]. Briefly, mice tissues were homogenized by BioMasher II (Nippi, Tokyo, Japan) in lysis buffer (50 mM Tris–HCl [pH 8.0], 2 mM DTT, 2 mM EDTA, and 1% Triton X-100). Tissue lysates were incubated in reaction buffer (100 mM Tris–HCl [pH 8.0], 100 mM KCl, 0.5 mM EDTA, 2 mM MgCl_2_, 0.2 mM NADH, 3 mM ADP, and 10 μM 2,3-diphosphoglycerate) with enzyme mixture (0.6 U lactate dehydrogenase, 0.5 U pyruvate kinase, and 0.1 U enolase). The levels of NADH were monitored at 37°C, from adding to 1 mM 3-phosphoglyceric acid. Enzymatic activity was measured as NAD^+^ release.

### RNA analysis

Total RNA was extracted with TRIzol (Invitrogen). cDNA pools were prepared by the ReverTra Ace qPCR RT kit (Toyobo, Osaka, Japan). Real-time quantitative PCR was carried out using Thunderbird SYBR qPCR mix (Toyobo). The level of expression of target gene was monitored by the *Thermal Cycler Dice Real-Time system* (Takara Bio., Kusatsu, Japan) *and*. Gene expression levels were normalized to *Rpl13a* mRNA and presented as values relative to wild-type mice.

The primer sequences are shown as below. *Rpl13a*:Fw 5’- TGC TGC TCT CAA GGT TGT TCG -3’, Re 5’-GCC TTT TCC TTC CGT TTC TCC-3’, *Hk1*:Fw 5’-AAG AAT GGC CTC TCC CGG-3’, Re 5’-CGC CGA GAT CCA GTG CAA TG-3’, *Hk2*:Fw 5’-ATA TGG TTG CCT CAT CTT GG-3’, Re 5’-CTC CCT CCC TCC CAA TG-3’, *Hk3*:Fw 5’-ATT CCT GGA TGC ATA CCC CGT -3’, Re 5’-GCC GCT GCA CCT AAA ACC TTT -3’, *Gpi*:Fw 5’-CCA ATG CAG AGA CAG CAA AGG-3’, Re 5’-CAC TTT GGC CGT GTT CGT AGA-3’, *Pfkl*:Fw 5’-GCT GCA ATG GAG TTG TG-3, Re 5’-GTA GCC AGG TAGC CAC AG-3’, *Pfkm*:Fw 5’-TGG AGC GAC TTG CTG AAT GAT -3’, Re 5’-TCA TTG TCG ATT GAG CCA ACC -3’, *Aldo a*:Fw 5’-CTG GCC ATC ATG GAA AAT GC-3’, Re 5’-TCA AGT CAT GGT CCC CAT CAG-3’, *Aldo b*:Fw 5’-ATC GGC GGA GTG ATC CTT TT-3’, Re 5’-TCC AAC TTG ATG CCC ACC A -3’, *Tpi*:Fw 5’-TGC CAA ACA ATG AGC ACT GC-3’, Re 5’-ATC AGA AGC ATG TGA CCG GTG-3’, *Gapdh*:Fw 5’-AGC CTC GTC CCG TAG ACA AAA-3’, Re 5’-TGG CAA CAA TCT CCA CTT TGC-3’, *Pgk1*:Fw 5’-TTT GGA CAA GCT GGA CGT GAA-3’, Re 5’-GCT TGG AAC AGC AGC CTT GAT-3’, *Pgam1*:Fw 5’-GTT GCG AGA TGC TGG CTA TGA-3’, Re 5’-CAC ATC TGG TCA ATG GCA TCC-3’, *Pgam2*:Fw 5’-TGG AAT GAG GAG ATC GCA CCT -3’, Re 5’-TCG GAC ATC CCT TCC AGA TGT -3’,*Eno1*:Fw 5’-TAT TGC GCC TGC TCT GGT TAG-3’, Re 5’-GGA TGG CAT TTG CAC CAA AT-3’, *Eno3*:Fw 5’-GGA GAA GAA GGC CTG CAA TTG -3’, Re 5’-CCC AGC CAT TAG ATT GTG CAA -3’,*Pkm1*:Fw 5’-CTG TTT GAA GAG CTT GTG GCG -3’, Re 5’-CTG CTA AAC ACT TAT AAG AGG CC -3’.

### Statistical analysis

All data are expressed as the mean ± standard error of the mean (SEM) Comparisons between two independent groups were analyzed using an unpaired Student’s two-tailed t-test.

## Results

### Homozygous knockout mice of *Pgam1* were embryonic lethal

**Fig 1:**
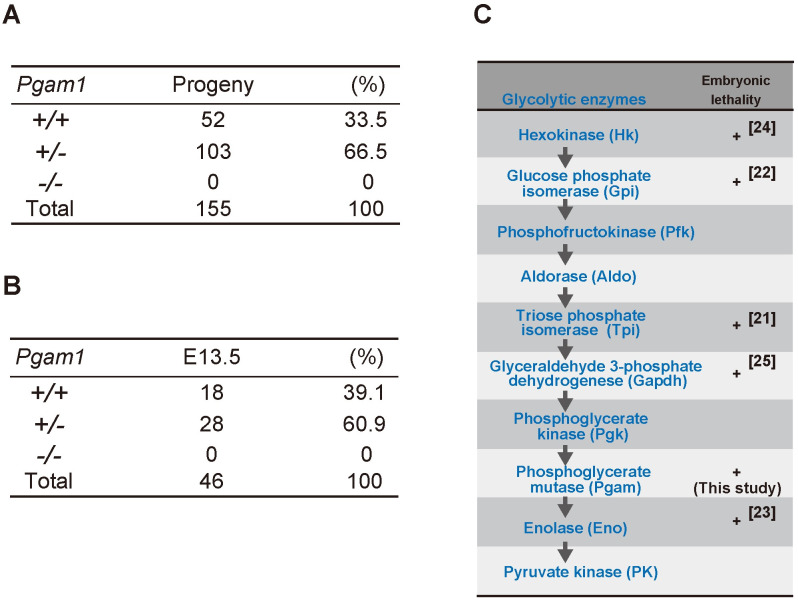
Embryonic lethality of homozygous knockout mice of *Pgam1*. **(A)** Summary of *Pgam1* genotypes for 155 progenies from crosses between *Pgam1*^*+/−*^ mice. +/+; wild-type, +/−; heterozygous KO, −/−; homozygous KO. **(B)** Summary of *Pgam1* genotypes for embryo from crossing between *Pgam1*^*+/−*^ mice at embryonic day 13.5 (E13.5). **(C)** Schematic diagram of glycolytic pathway. (+) indicates embryonic lethality in homozygous knockout mice, as reported previously (ref. [21–25]).

To analyze the physiological impact of PGAM inactivation *in vivo*, conditional KO mice for *Pgam1* were established [18]. Global *Pgam1* heterozygous KO (*Pgam1*^*+/−*^) mice were generated through crosses between *CAG-Cre* Tg mice and *Pgam1*^*flox/+*^ mice. Among 155 progenies obtained after mating between *Pgam1*^*+/−*^ mice, the ratio of wild-type; heterozygous KO; and homozygous KO was 52:103:0 (Fig 1A). Thus, *Pgam1* homozygous KO (*Pgam1*^*−/−*^*)* mice were embryonic lethal, consistent with the lack of observed *Pgam1*^*−/−*^ embryos at embryonic day 13.5 (E13.5) (Fig 1B). The early embryonic lethality of *Pgam1*^*−/−*^ mice validated the vital significance of PGAM *in vivo*, like the other glycolytic enzymes [21–25] (Fig 1C).

### Heterozygous *Pgam1* knockout mice were viable with reduced PGAM acitvity

**Fig 2:**
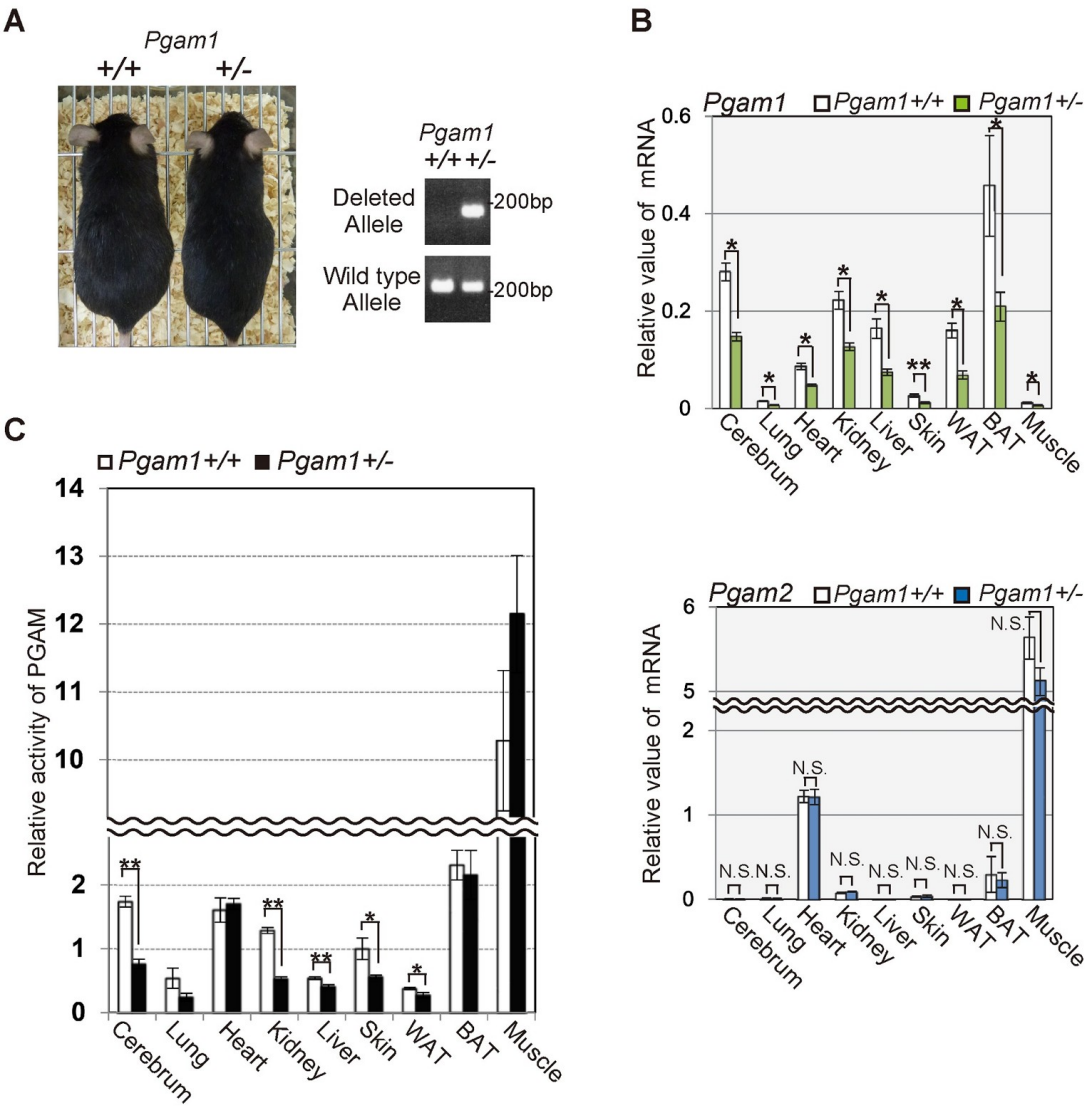
Heterozygous *Pgam1* knockout mice were viable. **(A)** *Pgam1*^*+/−*^ mice are viable. Representative images of wild-type and *Pgam1*^*+/−*^ mice at 35 weeks (left panel). Genomic PCR in indicated mice was performed to verify the wild-type or deleted alleles for *Pgam1* (right panels). **(B and C)** Assessment of PGAM expression levels and enzymatic activity in various tissues from wild-type (n = 5) and *Pgam1*^*+/−*^ mice (n = 5) at 35 weeks. **(B)** *Pgam1*^*+/−*^ mice displayed approximately 50% reduction for *Pgam1* mRNA levels, compared to those from wild-type mice (upper panel), while *Pgam2* mRNA levels did not show significant difference (lower panel). **(C)** The PGAM activity were measured in indicated tissues. Each data is shown as relative values against those in control. +/+; wild-type, +/-; heterozygous knockout.

Although *Pgam1* homozygous KO mice were embryonic lethal, *Pgam1*^*+/−*^ mice are viable (Fig 2A). The mRNA levels of *Pgam1* were decreased by approximately 50% in several tissues of *Pgam1*^*+/−*^ mice (Fig 2B, upper panel), whereas the levels of *Pgam2* mRNA were not affected in the examined tissues from these mice (Fig 2B, lower panel). PGAM1 and PGAM2 exhibit overlapping but distinct tissue distributions; PGAM1 is dominantly expressed in several tissues including the liver, white adipose tissue, aorta, and brain, whereas PGAM2 is found mainly in the muscle. The other tissues including the lung, heart, skin, and bone express both isoforms [11,13]. Consistently, the enzymatic activities of PGAM in *Pgam1*^*+/−*^ mice were significantly decreased in several tissues including the cerebrum, kidney, and skin, or modestly decreased in the lung, liver, and white adipose tissue (WAT), but not in the heart, brown adipose tissue (BAT), and muscle, compared to those in control (Fig 2C). As several posttranslational modification for PGAM is heavily operating [26], unknown compensatory mechanisms by such modification may preserve its enzymatic activity in *Pgam1* heterozygous knockout mice.

### *In vivo* parameters for glucose metabolism were less affected by heterozygous *Pgam1* knockoout

**Fig 3:**
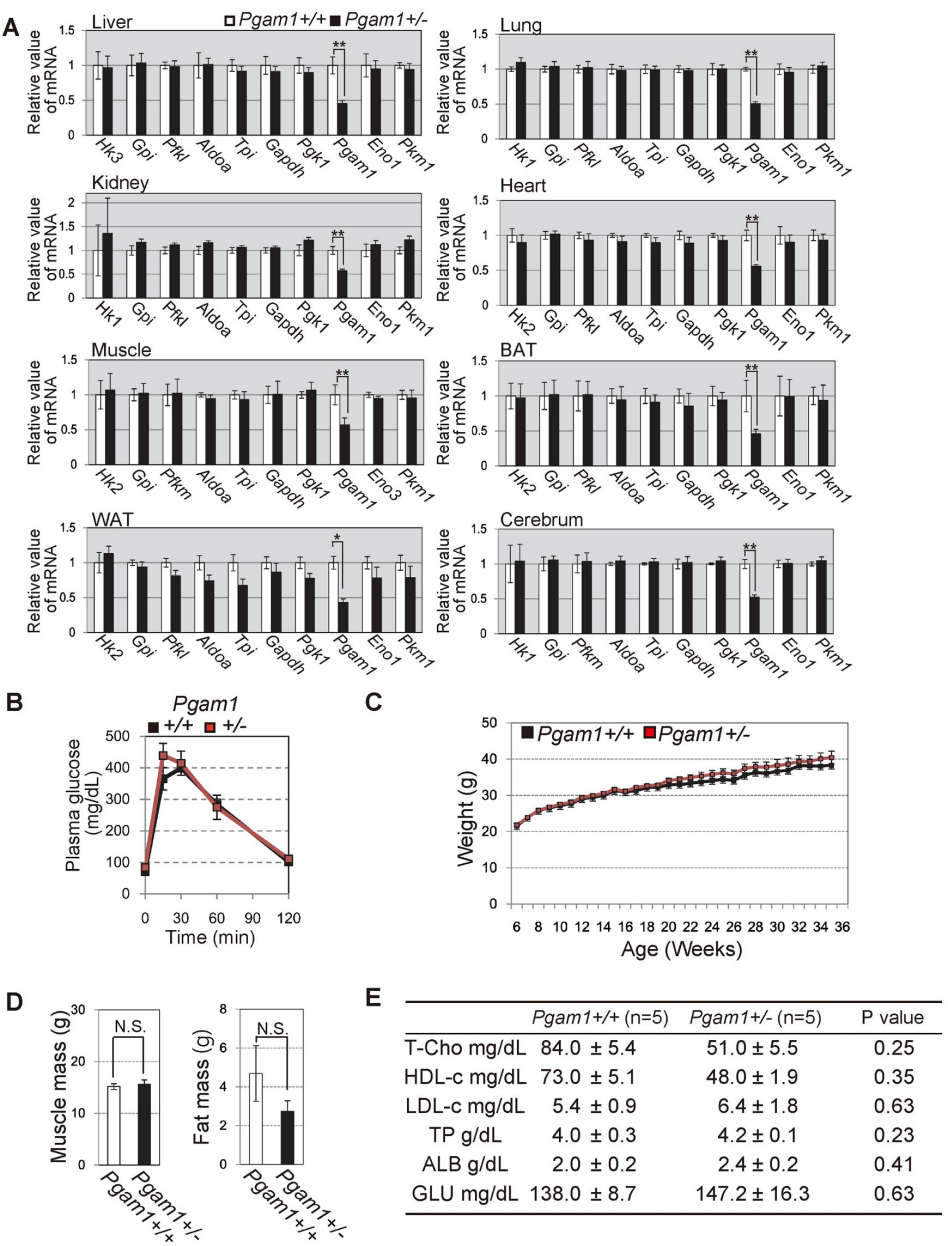
Characterization of *Pgam1* heterozygous KO mice. **(A)** The comparison of glycolytic mRNAs profiles between wild-type and *Pgam1*^*+/−*^ mice. The extracts from indicated tissues were tested. (B) Intraperitoneal glucose test (IPGTT) was performed in wild-type (n = 5) and *Pgam1*^*+/−*^ mice (n = 4). **(C-E)** Several physiological parameters in wild-type (n = 5) and *Pgam1*^*+/−*^ mice (n = 5). **(C)** Body weight were measured every week for 30 weeks. **(D)** Measurements of muscle mass and visceral-fat mass by computerized tomography at 35 weeks. **(E)** Total cholesterol (T-Cho), high-density lipoprotein cholesterol (HDL-c), low-density lipoprotein cholesterol (LDL-c), Total protein, (TP), albumin (ALB) and blood glucose (Glu) were determined in the blood plasma of 35 weeks mice. +/+; wild-type, +/-; heterozygous knockout. Data represent the mean ± SEM. Single-asterisks (*) and double-asterisk (**) indicate statistical significance of p<0.05 and p<0.005, respectively, Student’s t-test.

Recently we reported that PGAM status deeply affects glycolytic profiles in cancer cells with oncogenic Ras expression, but not in standard cells *in vitro* [18]. We examined the profiles of glycolytic mRNAs in the tissues of *Pgam1*^*+/−*^ mice. We observed no significant difference in the profiles of glycolytic mRNAs between wild-type and heterozygous knockout of *Pgam1* mice (Fig 3A).

It was reported that brain-specific knockout of Glut4 *in vivo*, glucose transporter in glycolysis, displayed impaired glucose tolerance and insulin sensitivity [27]. As glycolysis is essential energy source in all tissues, it is possible that the impaired glycolysis in the limited organs could affect glucose metabolisms *in vivo* [28]. To examine this possibility, we next examined glucose metabolism of *Pgam1*^*+/−*^ mice *in vivo*, as mice with one allele of glucokinase displayed diabetic phenotype [8]. In comparison to wild-type mice, *Pgam1*^*+/−*^ mice displayed similar profiles for *in vivo* glucose metabolism (intraperitoneal glucose tolerance test (IPGTT) and serum glucose level), in addition to body weight, visceral-fat weight, muscle mass, total-cholesterol, and other biochemical blood markers (Fig 3B–3E). Collectively, *in vivo* parameters for glucose metabolism were less affected by heterozygous *Pgam1* knockout.

### Global overexpression of *Pgam2* does not affect glucose tolerance *in vivo*

**Fig 4:**
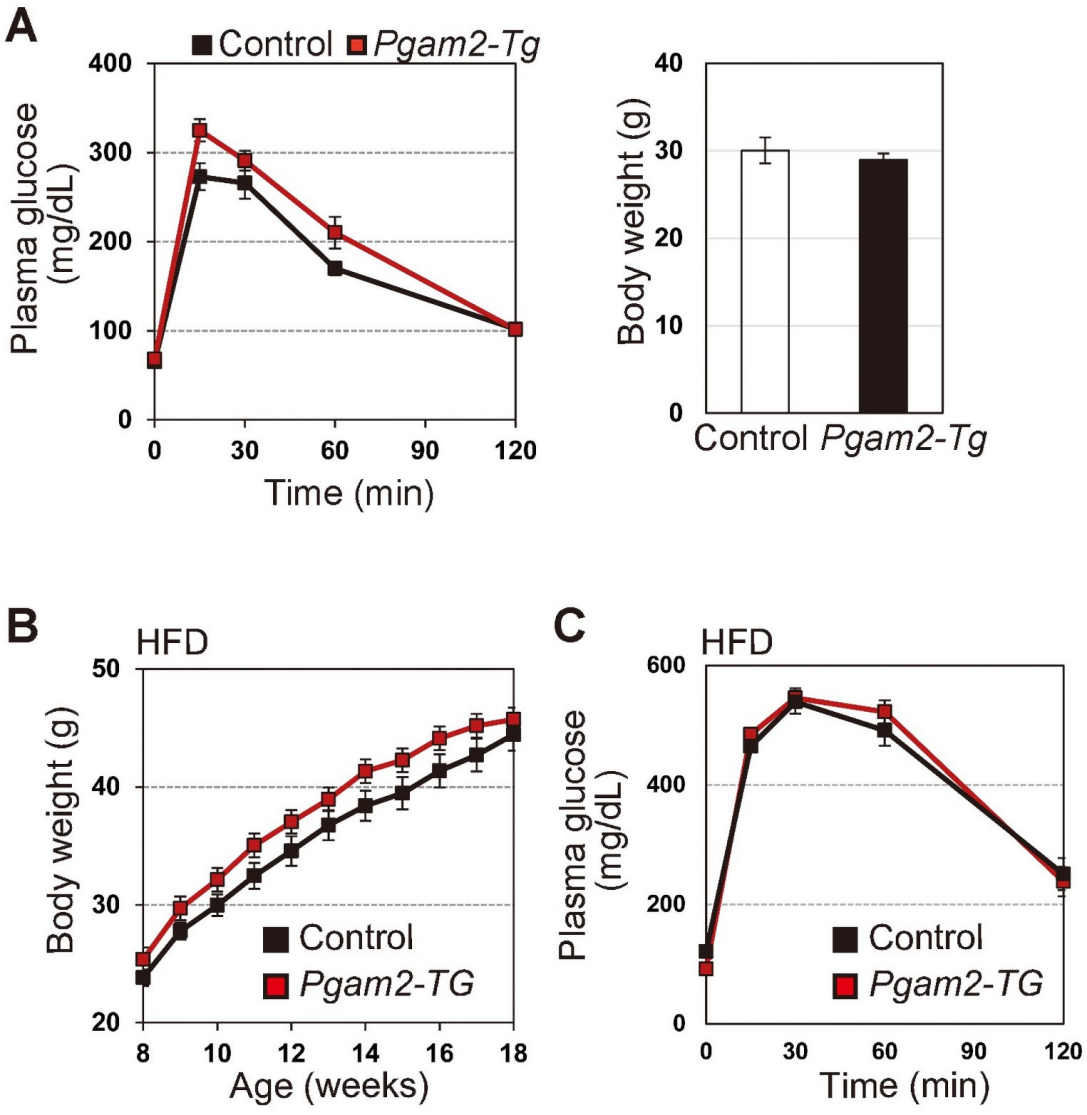
Glycolytic profiles in PGAM2-Tg mice. **(A)** Intraperitoneal glucose test (IPGTT) was performed in control (wild-type) (*n* = 10) and *Pgam2*-Tg mice (*n* = 8) (Left panel). Body weight was measured at 18 weeks (Right panel). **(B and C)** High-fat diet (HFD) protocol was performed to induce obesity in control (n = 9) and *Pgam2*-Tg mice (n = 7). **(B)** Body weight was monitored during the protocol (left panel). **(C)** IPGTT was performed in HFD-fed obese mice at 18 weeks (right panel). Data represent the mean ± SEM.

Next, we evaluated the impact of PGAM overexpression *in vivo*. We previously reported that heart-specific *Pgam2*-transgenic (Tg) mice displayed almost normal glycolytic features in the heart [16]. However, it remains unclear whether global PGAM overexpression affects glycolytic profiles *in vivo*. As it has been demonstrated that the overexpression of either PGAM isoform confers similar physiological impact [11–13], we evaluated the *in vivo* parameters of glucose metabolism in *Pgam2*-Tg mice, in which a *Pgam2*-FLAG transgene is driven by the *CAG* promoter. *Pgam2*-Tg mice exhibited a significant increase of PGAM protein in the whole body [18]. *Pgam2*-Tg mice also displayed their similar features with respect to body weight and IPGTT both under physiological (Fig 4A) and high fat-induced obesity conditions (Fig 4B and 4C), compared with those of wild-type mice. Thus, the parameters for glucose metabolism are less affected by PGAM overexpressing status *in vivo* under physiological conditions.

## Discussion

Here we reported on the profiles of *in vivo* glucose parameters in genetic models of glycolytic enzyme PGAM. Consistent to the findings in the ablation of other glycolytic enzymes, the embryonic lethality of homozygous knockout of *Pgam1* supports the notion that glycolysis is vitally essential metabolism *in vivo*. Unexpectedly, however, *Pgam1*^*+/−*^ and *Pgam2* -Tg mice displayed comparable profiles of *in vivo* glucose parameters, as far as we tested.

Several genetic mutations of PGAM with its impaired enzymatic activity, have been reported in human PGAM deficient patients [29–31]. However, little is reported on the link between human PGAM deficiency and diabetes, except one case report on the adult patient of PGAM deficiency (51 years old) accompanied with type 2 diabetes [32]. Thus, it is still controversial whether human PGAM deficiency causes diabetes or not. Here, we observed that the *in vivo* parameters for glucose metabolism are less affected in *Pgam1*^*+/−*^ mice. Moreover, *Pgam2* -Tg in normal and high-fat diet conditions displayed the normal glucose tolerance. However, as *Pgam1* ^*+/-*^ mice retained substantial PGAM enzymatic activity in several tissue including liver, muscle, WAT and BAT, it would be worthy to investigate tissue-specific *Pgam1* homozygous knockout or *Pgam2* knockout model in the future.

Different from our observations in genetic model mice for PGAM, several reports suggest that impaired glycolysis results in diabetes or impaired glucose metabolism both in human patients [4,5] and in knockout (KO) mice for the other glycolytic enzymes [8,9]. The discrepancy between our study and the inactivation of the other glycolytic enzymes could be possibly due to the different impact of individual glycolytic enzymes on glycolytic pathway. Interestingly, the catalytic reaction of PGAM is not rate-limiting step during glycolytic pathway under standard conditions, even when PGAM activity is reduced by approximately half of normal mean value both in human PGAM deficient patient [32] and in *Pgam1* heterozygous knockout mice (this study) [18]. As PGAM enzyme activity is most prominent among glycolytic enzymes [30], the residual PGAM activity may be sufficient to maintain glycolysis in human or mice PGAM impairment. Interestingly, overall glycolysis is much affected if majority of PGAM proteins were degraded via ubiquitin-dependent proteolysis of PGAM under senescence-inducing stress [13], rather consistent to the embryonic lethality of *Pgam1*^*−/−*^ mice (in this study). In this setting, PGAM would be rate-limiting in glycolysis. Moreover, we recently reported that the global enhancement of glycolytic profiles in cancerous cells, the Warburg effect, is provoked *in vitro* by the cooperation of PGAM with Chk1 kinase under oncogenic Ras expression [18]. Besides cancers, it is well known that the active proliferation of T cells after immunological stimuli or stress is accompanied by the enhanced glycolysis [33]. *Pgam1* deficient T cells are impaired in maintenance of such glycolytic enhancement, followed by immunological dysfunction [34]. Thus, the impact of PGAM on glycolytic regulation would be cellular- or tissue-context dependent. Indeed, the boosts of glyclolytic mRNAs were observed in *Pgam2*-Tg mice under chemical carcinogenic protocol [18], while those in *Pgam2*-Tg mice under standard conditions were comparable to control [18]. It is possible that the modulation of PGAM proteins would be required *in vivo* for its physiological impact, as several post-translational regulations of PGAM were reported *in vitro* [26].

Further investigation might provide valuable clue to understand the impact of PGAM on glucose metabolisms *in vivo*.

## Supporting information

S1 Data Mikawa et al raw data. (XLSX)
